# Starch bioengineering affects cereal grain germination and seedling establishment

**DOI:** 10.1093/jxb/eru107

**Published:** 2014-03-18

**Authors:** Shahnoor S. Shaik, Massimiliano Carciofi, Helle J. Martens, Kim H. Hebelstrup, Andreas Blennow

**Affiliations:** ^1^Department of Plant and Environmental Sciences, University of Copenhagen, Thorvaldsensvej 40, 1871 Frederiksberg C, Denmark; ^2^Department of Molecular Biology and Genetics, Aarhus University, Forsøgsvej 1, 4200 Slagelse, Denmark

**Keywords:** Amylase, barley, cereal, germination, grain, starch.

## Abstract

Cereal grain germination is central for plant early development, and efficient germination has a major role in crop propagation and malting. Endosperm starch is the prime energy reserve in germination and seedling establishment. In this study, it was hypothesized that optimized starch granule structure, and not only the endosperm starch content *per se*, is important for germination and seedling establishment. For that purpose, wild-type (WT), and specifically engineered degradable hyperphosphorylated (HP) starch and more resistant amylose-only (AO) starch barley lines were used. The transgenics showed no severe phenotypes and the WT and HP lines degraded the starch similarly, having 30% residual starch after 12 d of germination. However, the AO line showed significant resistance to degradation, having 57% residual starch. Interestingly, protein and β-glucan (BG) degradation was stimulated for both HP and AO lines as compared with the WT. At late seedling establishment stages, specific sugars were rapidly consumed in the AO line. α-Amylase activity was distinctly suppressed in both the HP and the AO lines. Pre-germination β-amylase deposition was low in the AO grains and β-amylase was generally suppressed in both HP and AO lines throughout germination. As further supported by scanning electron microscopy and histochemical analyses on grain and seedlings, it was concluded that inadequate starch granule deposition in combination with the suppressed hydrolase activity leads to temporal and compensating re-direction of starch, sugar, and protein catabolism important to maintain metabolic dynamics during grain germination and seedling establishment.

## Summary

Grain starch phosphorylation and amylose content affect germination and seedling establishment through the combination of direct effects on altered starch granule and molecular structure and indirect effects on amylase activities.

## Introduction

Germination of grain commences with the uptake of water. Once germination is initiated, the predominant endosperm reserves, starch, cell wall, and storage proteins, are mobilized by the action of hydrolytic enzymes which are synthesized in the aleurone layer and in the scutellum and secreted into the starchy endosperm of germinating barley grains. Starch is the main storage compound synthesized in barley endosperm and serves as the primary source of carbohydrate during germination and seedling growth. α-Amylase enzyme plays a primary role in native starch granule degradation, and its expression is controlled by both gibberellin (GA) and sugar demand/starvation. Sugar or carbon starvation activates the α-amylase promoter (Lu *et al.*, 1998). Depletion of sugar and other nutrients such as nitrogen and phosphate leads to the activation of regulatory mechanisms of amylases and a variety of other hydrolases, transporters, and transcription factors (Hong *et al.*, 2012), which activates synthesis of α-amylase and other hydrolytic enzymes in the scutellum and the aleurone layer. Sucrose and raffinose play a major role during barley grain germination. Raffinose and sucrose levels decline during germination, and sucrose levels increase post-germination as the sugars from the modifying endosperm pass through the scutellum to the growing embryo (MacLeod, 1957; Palmer, 1969). Glucose and maltose are produced as a result of hydrolysis in the endosperm. Trehalose helps to stabilize proteins that are activated during early germination (Sreenivasulu *et al.*, 2008). The cell wall polysaccharides are degraded, which allows enzymes to access the cell content. The protein mobilization leads to amino acid supply to the growing seedling and also acts as an alternative source for carbon during restricted supply of carbon (Perata *et al.*, 1997; Araújo *et al.*, 2011). Lipids disappear from the scutellum before germination is evident (Palmer, 1969).

Normal starch granules consist of ~25% mostly linear α-1,4-linked amylose and 75% α-1,4 backbone and α-1,6 branched amylopectin deposited as alternating amorphous and crystalline layers. Parallel double helices in the amylopectin align to organize the amylopectin in two different types of crystalline polymorphs: the A-type crystalline polymorph consisting of densely packed double-helical segments typical of cereal endosperm starches; and the B-type crystalline polymorph, typically found in tuberous and leaf starches, and containing a substantial amount of structured water. A third type, the C-type polymorph, is commonly found in, for example, legume seeds, and is a mixture of the A-type and the B-type polymorphs. In high amylose varieties, a single helical polymorph often complexed with lipids is found, termed the Vh-type polymorph (Damager *et al.*, 2010; Pérez and Bertoft, 2010). The organization of amylose in the granule is complex (Glaring *et al.*, 2006) and its effect on amylopectin packing at low and moderate levels of amylose (Bocharnikova *et al.*, 2003; Kozlov *et al.*, 2007), and at high amylose levels (Bocharnikova *et al.*, 2003) are very different. The starch granule surface is the initial substrate for amylases. Such surfaces as studied by, for example, atomic force microscopy (Park *et al.*, 2011) indicate that so-called blocklet structures, which differ tremendously for different starch types, can be important for initial amylase binding and attack.

Starch molecular structures can be quite specifically bioengineered in the plant using mutagenesis and transgene technologies, especially in cereals (Blennow *et al.*, 2013), providing starch types with different physical states and surface boundaries to be tested for hydrolase interaction and catalysis. It has been demonstrated that the degradability of, for example, high-amylose and B-type starches is generally more resistant to amylolytic attack as compared with A-type starches (Planchot *et al.*, 1997; Jane *et al.*, 2003). Hence, amylopectin-only (wx) maize and normal maize granules were degraded similarly while high-amylose maize was more resistant towards fungal α-amylase (Planchot *et al.*, 1995). The importance of amylose for combined gluco-amylase and α-amylase resistance for an amylose-only barley model was recently demonstrated (Carciofi *et al.*, 2012). A crystalline polymorph *per se* is not important for degradability and, for lotus C-type starch granules, α-amylase did not selectively digest A- or B-type polymorphs in this starch (Lin *et al.*, 2006). Moreover, no major preferential degradation of starch amylopectin or amylose could be noted during ripening in banana (Soares *et al.*, 2011). However, a slight preferential degradation of amylose was noted for legume starches (Zhou *et al.*, 2004). However the precise basis for these effects has not been fully explained (Englyst *et al.*, 1992). It has been proposed that granule surface topography and crystal defects influence degradation (Jane *et al.*, 2003). For example, degradation of A-type maize starch showed initial attack at susceptible surface areas followed by pore formation (Planchot *et al.*, 1995). The starch granule surface area and the degree of molecular order in the starch granule as measured by differential scanning caloriometry (DSC) are possibly important for enzymatic efficiency with pancreatic α-amylase (Tahir *et al.*, 2010). However, molecular disorder is not the only parameter and with extreme molecular disorder and, in high-amylose and amylose-only type starches that have extreme molecular disorder, hydrolytic efficiency is severely restricted *in vitro* (Carciofi *et al.*, 2012). Accordingly, for the maize starch system, evidence was provided that hydrolytic sensitivity is accounted for by long-range structural effects controlling enzyme diffusion to starch granule surfaces (Shrestha *et al.*, 2012).

Tuberous starches are highly phosphorylated by glucan water dikinases (GWDs) providing weak spots in the starch granule to support cellular starch degradation by local hydration and amorphization (Damager *et al.*, 2010; Blennow and Engelsen, 2010). These phosphate groups open up and weaken sections in the starch granule to stimulate hydrolytic attack. To the authors’ knowledge, there has been no evidence on *in vivo* effects of starch bioengineering relating to starch granule architecture on cereal grain starch metabolism during germination and seedling establishment. Using transgenic barley lines specifically affected in starch granule architecture, it is demonstrated here that correctly structured endosperm starch granules are vital for their bioavailability/catabolism, establishing correct initiation and maintenance of metabolic dynamics during grain germination and seedling establishment, whereas inadequate granule structure leads to reduced starch hydrolysis and compensating re-directions of starch, sugar, and protein catabolism and hence primary metabolism of the growing seedling.

## Materials and methods

### Grain material and grain biomass remobilization

Grains of the wild type (WT) barley cultivar Golden promise, the hyperphosphorylated (HP; *Solanum tuberosum* GWD overexpressor, Carciofi *et al.*, 2011), and the amylose-only [AO; starch branching enzyme RNA interference (RNAi) suppressor, Carciofi *et al.*, 2012] line were germinated in sealed plastic containers on filter paper moistened with autoclaved double-distilled water (ddH_2_O) for 4, 8, and 12 d. It is important to note that all of the three types of grains that were compared originate from the same inbred cultivar. The germination was conducted in the dark in a CMP 6010 growth cabinet (Conviron Ltd, Winnipeg, Canada) at a constant temperature of 23 °C. After germination, the emerging parts, the coleoptiles and radicles, were excised from the grains. Each part was dried for 3 d at 95 °C. The dry weight was determined on a standard laboratory scale to calculate the amount of biomass that has been remobilized from each grain.

### Total starch content

Barley pre-germinated dry grain and seedlings germinated for 4, 8, and 12 d were used for analysis. Coleoptiles and radicles (if present) were cut off and residual grains were freeze-dried. Freeze-dried residual grain samples were ground in a tissue lyser (TissueLyser, Retsch, Qiagen) operating at 30 Hz for 15 s and passed through a 0.5mm screen. Flour samples of 10mg each were used for analysis. Soluble sugars were removed by washing the flour in 80% (v/v) ethanol, in a boiling water bath for 30min and once for 10min, and in 96% ethanol at room temperature for a final wash. The supernatant was removed at the end of every step after centrifugation at 9500 *g* (Heraeus biofuge pico, Thermo, Germany) for 5min and the pellet was dried at 40 °C. The pellet was re-suspended using solutions of 2M KOH and 1.2M sodium acetate buffer (pH 3.8) according to the protocol described by the manufacturer (AOAC Method 996.11/AACC Method 76.13); the reaction volumes were scaled down according to the smaller sample weight from Megazyme International Ltd (Wicklow, Ireland) following the protocol recommended by the manufacturer for samples containing resistant starch.

Total starch was completely hydrolysed by incubation of the samples for 6h at 80 °C with 10 μl of heat-stable α-amylase (Termamyl, Novozymes, Denmark) corresponding to 1.5 KNU (Kilo Novo Units), followed by incubation at 60 °C overnight with 10 μl of amyloglucosidase (Dextrozyme, Novozymes, Denmark) corresponding to 3.0 AGU (Amyloglucosidase Units). The whole reaction was conducted by continuous shaking at 900rpm in a thermomixer (Eppendorf Thermomixer Comfort, Germany). Samples were prepared for chromatography by centrifugation and filtration, and the supernatant was analysed with a high performance anion exchange system (Thermo Fisher Scientific Inc., Sunnyvale, CA, USA) with an AS50 autosampler, GS50 gradient pump, and ED50 PAD system equipped with a CarboPac^®^ PA1 column (Blennow *et al.*, 1998).

### Protein content

Total nitrogen was determined on finely ground barley pre-germinated dry grain and seedlings using an ANCA-SL/GSL elemental analyser (SerCon, UK), the technique that uses the Dumas procedure for the preparation system, and a stable isotope mass spectrometer. Nitrogen values were multiplied by a factor of 5.45 (Mariotti *et al.*, 2008) to give protein values.

### Soluble sugars

Barley pre-germinated dry grain and seedlings germinated for 1, 2, 3, 4, 8, and 12 d were used for analysis. Coleoptiles and radicles (if present) were cut off and residual grains were freeze-dried. Freeze-dried residual grain samples were ground in a tissue lyser (TissueLyser, Retsch, Qiagen) operating at 30 Hz for 15 s and passed through a 0.5mm screen. A 10mg aliquot of each flour sample was extracted in 80% (v/v) ethanol at 80 °C for 2h in a thermomixer. The samples were centrifuged for 5min at 13 000 *g* and the supernatant collected. The samples were then re-extracted in 80% (v/v) ethanol for 30min, centrifuged, and the supernatant was collected and pooled with the one from the previous extraction, and dried in a spin-vac. The samples were then reconstituted in water to the correct dilutions for analysis. Soluble sugars were identified and quantified using a gradient high performance anion exchange system (Thermo Fisher Scientific Inc.) with an AS50 autosampler, GS50 gradient pump, and ED50 PAD system equipped with a CarboPac^®^ PA1 column.

### *β*-Glucan content

A 10mg aliquot of flour samples was used for analysis. Soluble sugars were removed by washing the powder in 80% (v/v) ethanol, in a boiling water bath for 30min and once for 10min, and 96% ethanol at room temperature for a final wash. The supernatant was removed at the end of every step after centrifugation at 16 000 *g* (Heraeus biofuge pico) for 5min and the pellet was dried at 40 °C. Complete hydrolysis of BGs was conducted using lichenase and β-glucosidase from the mixed-linkage BG kit (Megazyme International Ltd) following the method provided by the manufacturer (AOAC Method 995.16 AACC Method 32-23/ICC Standard Method 32-23 No. 168; the reaction volumes were scaled down according to the smaller sample mass). Samples treated in that way were centrifuged, filtered, and the quantification of the resulting glucose units was performed by a high performance anion exchange system (Thermo Fisher Scientific Inc.) with an AS50 autosampler, GS50 gradient pump, and ED50 PAD system equipped with a CarboPac^®^ PA1 column using the run program described before (Lunde *et al.*, 2008).

### Enzyme activity assay

The α- and β-amylase activities were measured using enzymatic assay kits for these individual enzymes purchased from Megazyme International Ltd. The enzyme assays were performed on 5mg of flour samples and the reaction volumes were scaled down according to the smaller sample weight. For the β-amylase activity, cysteine was added in the extraction buffer to extract the insoluble β-amylase to provide ‘total’ β-amylase activity (as per Megazyme’s protocol).

### Isolation of starch

Starch was extracted and purified from 0.5g of malt flour using a modified version of the protocol described by Carciofi *et al.* (2011). The residue after SDS and dithiothreitol (DTT) treatment was subjected to enzyme treatments, 2.5U of proteinase K (Sigma-Aldrich) and 0.1U of β-glucanase from Megazyme International Ltd, to remove protein and cell wall debris. Samples were digested overnight and filtered through a 100 μm pore size nylon filter. Crude starch slurry was centrifuged for 10min at 4000 *g* (Allegra X-22R centrifuge, Beckman Coulter, Germany). The precipitate was suspended in 200 μl of water, layered over 1ml of 80% (w/v) caesium chloride solution, and centrifuged at 13 000 *g* (Allegra X-22R centrifuge, Beckman Coulter) for 15min. The starch pellet was washed twice with water, followed by acetone washing and overnight air drying.

### Amylose content by iodine complexation

The amylose content was determined by iodine colorimetry (Wickramasinghe *et al.*, 2009).

### Scanning electron microscopy

Granule morphology during germination was studied by scanning electron microscopy (SEM) as described (Carciofi *et al.*, 2011).

### Histochemistry

Barley pre-germinated dry grain and seedlings germinated for 4, 8, and 12 d were prepared for light microscopy. The glumes/husks were removed and the grains were trimmed with a razor blade in order to aid fixation and infiltration of resin. Grains and seedlings were fixed at their full length for 24h in Karnovsky’s fixative [5% (w/v) glutaraldehyde, 4% (w/v) paraformaldehyde, 0.1M sodium cacodylate buffer] including a 1h vacuum treatment, washed in cacodylate buffer at pH 7.3, and post-fixed in 1% (w/v) osmium tetroxide (with 0.1M cacodylate buffer) for 8h at 4 °C. After washing in buffer and water, the samples were dehydrated in a graded acetone series, infiltrated with three different ratios of Spurr resin to acetone, and embedded in Spurr resin within flat moulds. The resin was polymerized in an oven at 60 °C for 8h. Semi-thin section of 2 μm were cut with a histo-diamond knife on a Reichert–Jung supernova ultramicrotome and stained with periodic acid–Schiff’s (PAS) for insoluble carbohydrates containing 1,2-glycol groups and with 1% (w/v) amido black in 7% (w/v) acetic acid for proteins. Sections were viewed in immersion oil in a Nikon Eclipse 80i light and fluorescence microscope. Contrast adjustments were carried out to improve clarity of images but did not alter the overall appearance. Final image processing, cropping, and mounting of the images were done with Adobe Photoshop CS2 and Illustrator CS2.

### Statistical analysis

The statistical significance tests were performed using the one-way analysis of variance (ANOVA) statistical tool embedded in SigmaPlot 12.0. The term significant is used in the text only when the change has been confirmed to be significant (*P*<0.05/0.01/0.001) compared with the WT.

## Results

**Fig. 1.**
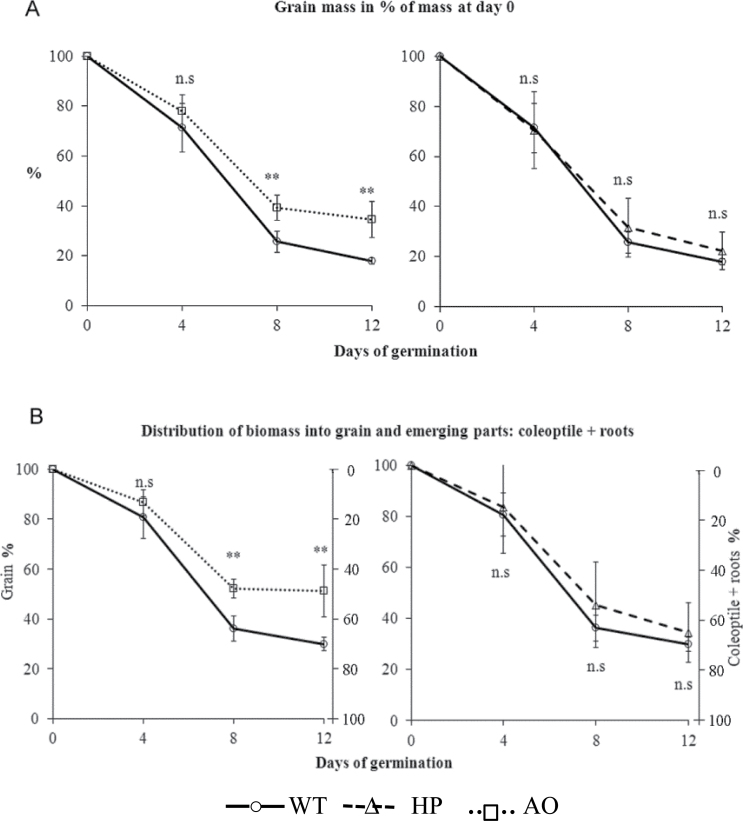
Remobilization of biomass (dry weight) during germination and seedling establishment of grains (dry weight). (A) Grains lose mass during germination. (B) Biomass is mobilized from grains into the emerging plant parts, the coleoptile and roots. The distribution of biomass between the grain and the emerging parts was determined during germination. Student’s *t*-tests were performed to test for difference between WT, AO and HP grains. n.s, not significant; **P<0.01; *n*=20 for WT and AO grains; *n*=10 for HP grains. Bars indicate standard deviations.

Grain serves as storage for biomass for remobilization in the next generation. In cereal grains, the majority of this storage compound is starch. The effect of the bioengineering of the two *in planta* modified starch types AO (Carciofi *et al.*, 2012) and HP (Carciofi *et al.*, 2011) on biomass remobilization in barley grains was tested by germinating them in ddH_2_O in the dark (Supplementary Fig. S1 available at *JXB* online), where the grain is the only carbon source. During a period of 12 d after imbibition, which was divided into four stages, germination (day 1), early stage of seedling establishment (day 2 to day 4), mid stage of seedling establishment (day 4 to day 8), and late stage of seedling establishment (day 8 to day 12), the dry mass of the remaining grain and the mass of the emerging parts—coleoptile and radicle—to which some of the original biomass had been remobilized, was measured. Less dry mass was remobilized from the grains containing AO starch, which represents a more resistant starch type (Carciofi *et al.*, 2012), than from grains with a control starch type (Fig. 1A, left). Also, grains with the AO starch type were less effective in remobilizing the biomass into the emerging parts of the new plant (Fig. 1B, left); and at 12 d post-germination in the dark, ~50% of the total remaining dry biomass was still in the grain of high-amylose grains, whereas >70% had been remobilized into emerging coleoptiles and roots in control grains. A similar effect was not significant for grains with the HP starch type (Fig. 1A, B, right).

Starch is the main source of carbon during germination and seedling establishment. The impeded remobilization of biomass from grains to plantlets in the AO grain type suggests that optimally structured endosperm starch granules are required for normal starch mobilization and hydrolysis. It was previously shown that purified starch from the AO line has a higher resistance to *in vitro* amylolytic degradation (Carciofi *et al.*, 2012). This shows that inadequate structures can limit starch degradation and hence germination and seedling establishment despite sufficient amount of endosperm starch in the grain. To assess the specific effects of starch structure and composition on barley grain germination dynamics and seedling establishment, grain internal morphology was investigated with microscopy, and key constituents including starch, protein, BG, sugar profiles, and amylolytic enzyme activities were quantified as a function of the germination time course.

### Starch degradation

**Fig. 2.**
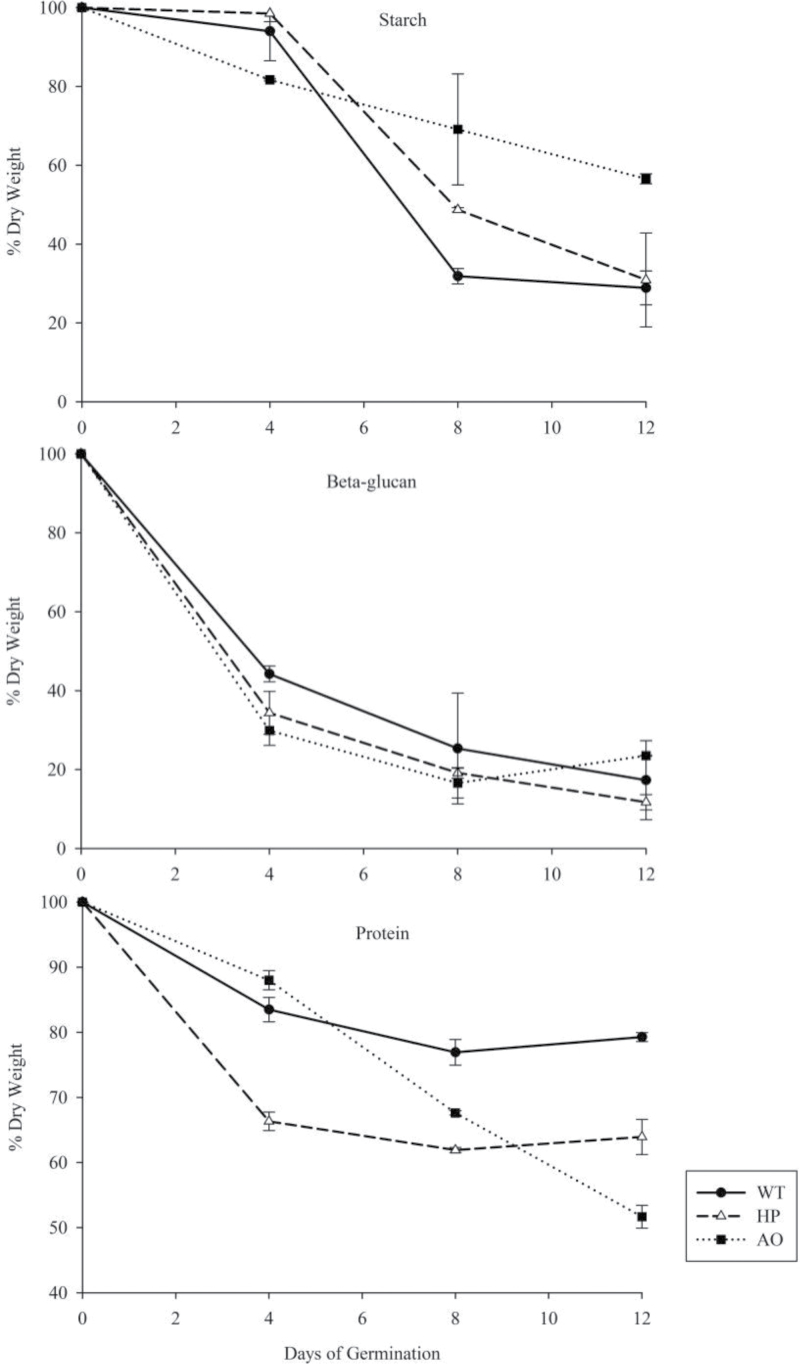
Remobilization of grain reserve components during germination and seedling establishment of grains (dry weight). Relative content of starch, BG, and protein in pre-germinated dry grain, and 4, 8, and 12 d germinated seedlings of the WT (open circles with solid line), HP (open triangles with dashed line), and AO lines (open squares with dotted line) during germination. All the values are calculated based on their respective pre-germinated dry grain sample which has been normalized to 100%. Values are the mean±SE of three replicates. The actual values are given in Table 1. Values and statistical significance are presented in Supplementary Table S1 available at *JXB* online.

The initial contents of starch, protein, and BG in the dry grain are given in Table 1. The mobilization rates of starch during germination and seedling establishment were distinctly different between the three lines (Fig. 2, upper panel; Supplementary Table S1 available at *JXB* online). During germination on day 1 and the early stage of seedling establishment, starch content in the WT and the HP line showed almost no reduction, whereas the AO line showed a slow but considerable decrease in starch content. Starch content started to decline markedly at the end of the early stage of seedling establishment in the WT and HP line, but the AO line showed a slower, linear reduction in starch content, demonstrating loss of normal dynamics of starch mobilization. Starch mobilization from HP grain was slower than from the WT, but followed a similar sigmoidal pattern in the time course, ending with almost 30% of residual starch, while the AO line showed persistent reduction, with almost 57% of residual starch. Thus, the AO line efficiently degraded starch initially but both the transgenic lines showed restricted starch degradation, occurring at two different phases: in the HP line during the early stage of seedling establishment and in the AO line at mid and late stages of seedling establishment.

**Table 1.** Pre-germinated dry grain starch, BG, and protein content (% dry weight) of the WT, HP, and AO linesStarch, BG, and protein contents were determined on the pre-germinated dry grains.

Line	Starch	β-Glucan	Protein
Wild-type (WT)	50.02±8.86	5.91±0.01	10.50±0.67
Hyperphosphorylated line (HP)	59.00±13.64	5.41±0.01	11.84±0.11
Amylose-only line (AO)	48.03±13.48	6.64±0.11	12.04±0.25

Values (% dry weight) are presented as the means±SE of three replicates.

### Alternative carbon sources

#### *β*-Glucan

To investigate further if the differential rates of starch mobilization in the lines re-directed the metabolism to be compensated by another carbon source, the second possible source of carbon, namely BG, was next analysed (Fig. 2B; Supplementary Table S1 available at *JXB* online). Sugars resulting from cell wall dissolution contribute energy for the young seedling (Fincher, 2011). BG contents of WT, HP, and AO lines showed a similar and rapid decrease during germination and the early stage of seedling establishment, and declined more gradually in the mid and late stages of seedling establishment. Hence, BG does not seem to compensate for the missing carbon remobilization from starch in the AO line.

#### Protein mobilization

To investigate further if the differential rates of starch mobilization in the lines re-directed the metabolism to be compensated by another carbon source, the third possible source of carbon, namely, protein, was next analysed. Protein degradation provides the major direct source of amino acids to be directed to translation of vital hydrolytic enzymes for the growing embryo (Bewley, 1997). Protein may also provide carbon to compensate the restricted starch degradation with protein degradation, in the transgenic lines. The HP and WT lines showed similar trends in protein decline during germination and seedling establishment. However, the HP line showed a dramatically steeper initial decline as compared with the WT, with 64% of residual protein by the end of the late stage of seedling establishment when compared with 79% of residual protein in the WT (Fig. 2C; Supplementary Table S1 available at *JXB* online). The AO line showed a linear and gradual decrease in protein levels, with a higher reduction of 52% by the end of the late seedling establishment stage. Hence, the AO and HP lines both showed compensation of restricted carbon availability with protein mobilization.

### Soluble sugars

**Fig. 3.**
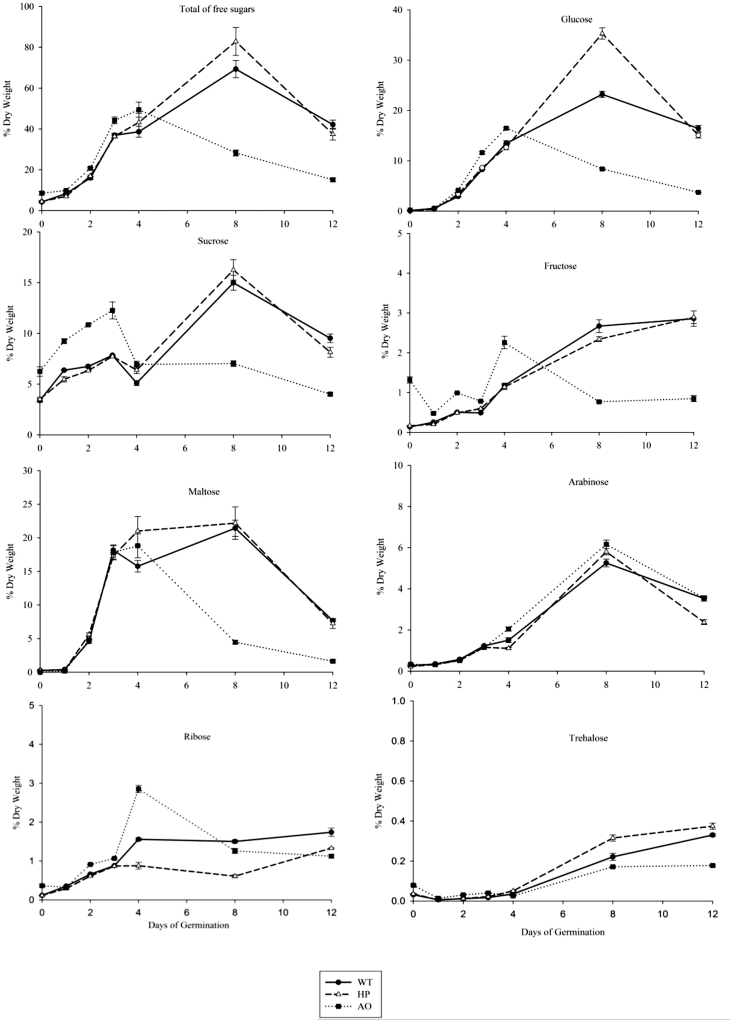
Changes in concentration of soluble sugars during germination and seedling establishment. Soluble sugars were measured in pre-germinated dry grain, and 4, 8, and 12 d germinated seedlings of WT (filled circles with solid line), HP (open triangles with dashed line), and AO lines (filled squares with dotted line). Values are the mean±SE of three replicates. Values and statistical significance are presented in Supplementary Table S2 available at *JXB* online.

Given the changes in the contents of starch and protein as carbon sources, the levels of soluble sugars representing glucose, fructose, sucrose, arabinose, trehalose, ribose, and maltose were next evaluated (Fig. 3; Supplementary Table S2 available at *JXB* online). Total soluble sugar was determined as the sum of the soluble sugar values. In order to obtain information on early metabolism during germination and throughout seedling establishment, the sugars that are either present in the pre-germinated dry grain or resulting from starch and cell wall breakdown were measured in dry grains at 1, 2, 3, 4, 8, and 12 d of germination and seedling establishment. The most striking differential effect in sugar dynamics for the three lines was the general decline in total sugars measured in the AO line as compared with the WT and HP line at mid and late stages of seedling establishment. During germination and the early stage of seedling establishment up to day 3, significant differences were observed for sucrose, fructose, and ribose, where the AO grain had higher levels of sucrose and fructose compared with the WT and HP grain; however, their levels then declined in the AO line in the mid and late seedling establishment stages. Substantial differences were also observed in glucose levels in mid and late stages of seedling establishment, where HP grain showed higher levels than the WT at day 8 (Fig. 3; Supplementary Table S2 available at *JXB* online). There were some differences in the levels of ribose especially in the seedling establishment stage among the WT, HP, and AO lines, indicating differences in nucleotide metabolism. There was no difference in arabinose levels between the WT, HP, and AO lines, indicating no differences in cell wall dissolution and also supporting the observation with the BG levels. Trehalose, which helps to stabilize proteins activated during early germination, showed no differences during germination and the early stages of seedling establishment. In general, all the sugars decreased rapidly at the late stage of seedling establishment, except fructose, ribose, and trehalose. During the late stage of seedling establishment, fructose, ribose, and trehalose showed a gradual increase in the WT and the HP line but remained constant in the AO line.

### Development of hydrolytic capacity over germination and seedling establishment

**Fig. 4.**
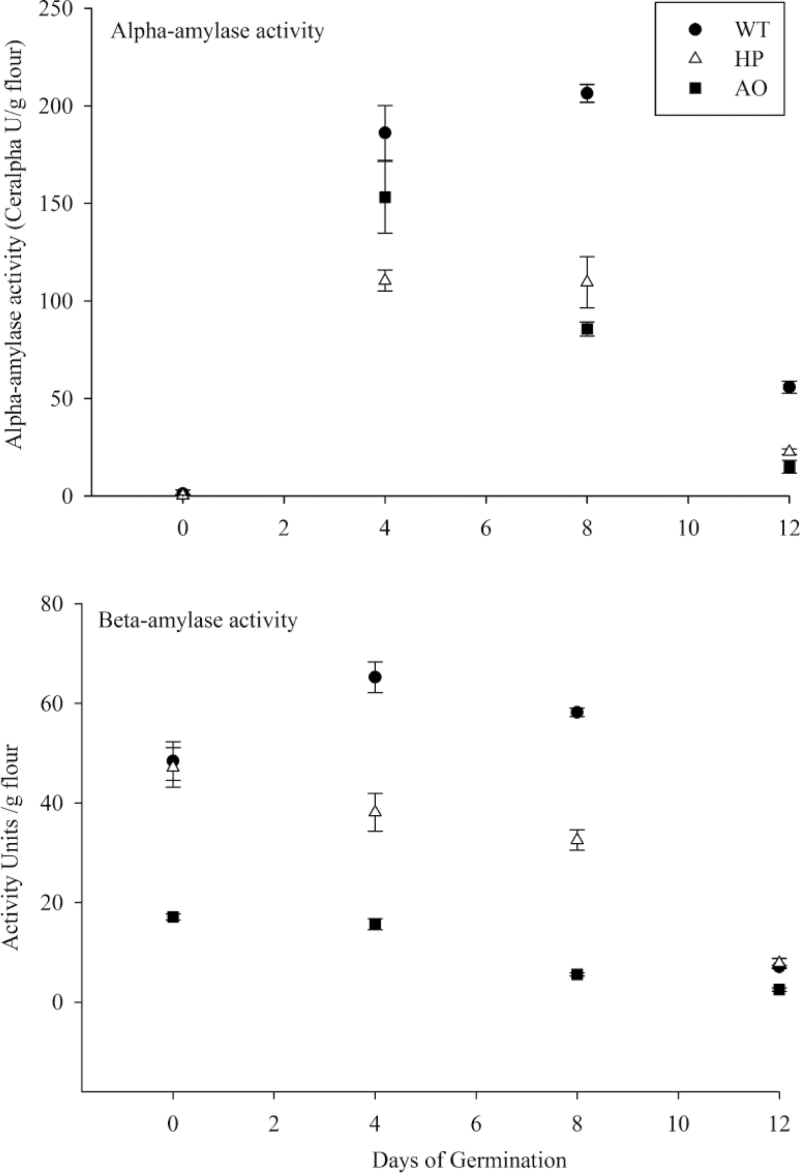
Time course of amylolytic enzyme activities in germinating barley grain extracts. Enzyme activities were measured in pre-germinated dry grain, and 4, 8, and 12 d germinated seedlings of WT (filled circles with solid line), HP (open triangles with dashed line), and AO lines (filled squares with dotted line). Values are the mean±SE of three replicates. Values and statistical significance are presented in Supplementary Table S1 available at *JXB* online.

α-Amylases are abundant and play a central role in the mobilization of starch during germination and seedling establishment (Smith *et al.*, 2005; Hong *et al.*, 2012). No α-amylase activity was detected in the pre-germinated dry grains of the WT, HP, and AO lines (Fig. 4, upper panel). After onset of imbibition, α-amylase activity increased rapidly but generally showed lower activity in the HP and AO lines when compared with the WT. Also in AO grain, activity declined earlier. In the WT and HP lines, the activity started to decline only at the end of the mid stage of seedling establishment.

β-Amylases play a more limited role in native starch degradation than α-amylase (Dunn, 1974). The initial total β-amylase activities were the same in the WT and HP lines, whereas the AO line had very low initial total activity (Fig. 4, lower panel). The AO line with lower total activity in the pre-germinated dry grain showed a nearly linear, non-dynamic decline in the levels of total activity throughout germination, and the early and mid stages of seedling establishment. The activities in the AO line declined to very low levels at the end of the germination period. HP grain showed a gradual decline until the end of the mid stage of seedling establishment and lower levels by the end of the late stage of seedling establishment. Interestingly, both transgenic lines had lower total β-amylase levels throughout the time course as compared with the WT. For both α-amylase and total β-amylase, the transgenic lines showed lower hydrolytic capacity and they both differed in the pattern of activity over the time course as compared with the WT.

### Glucan chain hydrolysis measured by iodine complexation

**Fig. 5.**
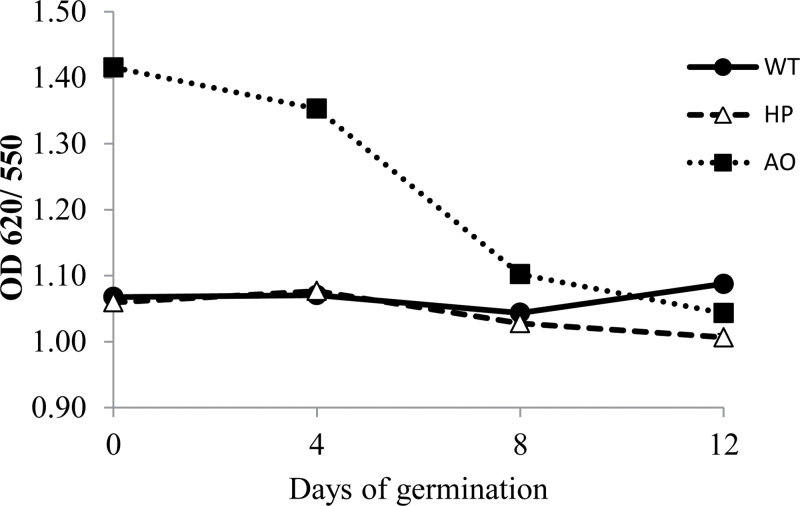
Glucan chain hydrolysis measured by iodine complexation of starch purified from pre-germinated dry grain, and 4, 8, and 12 d germinated seedlings of WT (open circles with solid line), HP (open triangles with dashed line), and AO lines (open squares with dotted line). Values are the mean±SE of three replicates.

Iodine complexation is an useful method for detecting the content of linear α-1,4 glucan chains in a starch-containing sample. These glucan segments can be free or linked by α-1,6 branch linkages such as in amylopectin, and their lengths distinctly determine the spectra of the iodine complexes formed, where long segments stain deep blue and blue-green, and shorter chains stain more weakly, having purple, red, and faint red colours with decreasing lengths (Bailey and Whelan, 1961). The optical density 620nm/550nm ratio (OD 620/550) was measured to assess the relative content of longer chain segments over the germination course. For the WT and HP lines, the OD 620/550 was constant throughout the entire course of germination while for the AO line there was a decline in this ratio (Fig. 5). OD 620/550 data can be interpreted as the apparent amylose content for a given starch. However, for an AO system, such data indicate the lengths of the nearly linear amylose chains, where high values indicate the presence of long chains. The constant OD ratio for the WT and HP lines indicates that neither amylose nor amylopectin was preferably degraded throughout germination. For the AO line, the decline indicates an attempt at multiple hydrolytic attack on the disordered starch granules in this line to generate numerous linear fragments with lower OD 620/550 values. To test if there was generation of such glucan fragments in the AO line, the grain content of malto-oligosaccharides was analysed using thin-layer chromatography (TLC) (Supplementary Fig. S3 available at *JXB* online) which was carried out as described in the Supplementary protocol for Supplementary Fig. S3 (available at *JXB* online). A distinct tailing was observed on the TLC plate in the day 8 and day 12 samples of AO, which was not present in the WT and the HP lines, supporting the presence of short chain glucans in the residual AO grain generated by multiple attacks on the rough surfaces of the AO starch granules.

### Scanning electron microscopy

**Fig. 6.**
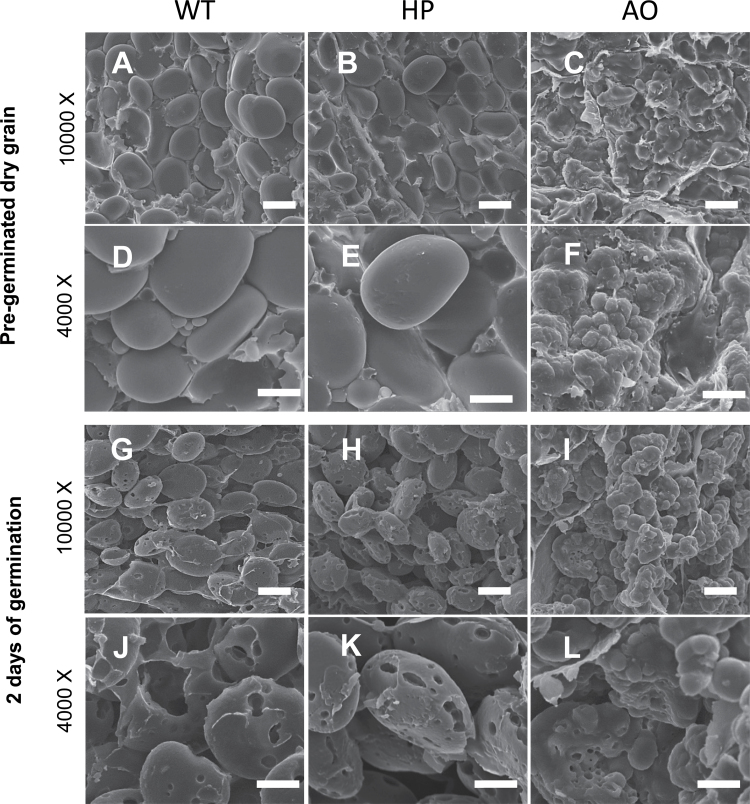
Scanning electron photomicrograph of a cross-section of a barley grain and seedling depicting starch granule degradation at the embryo–endosperm junction. Pre-germinated dry grain at ×4000 magnification (A–C) and at ×10 000 magnification (D–F). 2 d of germination at ×4000 magnification (G–I) and at ×10 000 magnification (J–L). Scale bars of 2 μm and 10 μm are indicated for ×4000 and ×10 000 magnification, respectively. A, D, G, and J are from the WT; B, E, H and K are from the HP line; and C, F, I, and L are from the AO line.

Longitudinally fractured halves of grains and seedlings of the three different lines were studied by SEM in order to investigate if the differences in compositional changes found between the three barley starch types could be effects of a differential degradation pattern of the starch granules and other grain components. Differences in starch granule topography and morphology are expected to be decisive for starch degradation since pores induced by high phosphorylation in the HP line and rough surfaces provided by non-structured amylose in the AO line will provide larger surface areas for hydrolytic attack. However, high amylose and amylose-only are known to restrict degradation (Carciofi *et al.*, 2012). Fractured longitudinal sections of dry and 2 d germinated barley grain, at the embryo–endosperm junction, in the WT, HP, and AO lines showed distinct differences in degradation patterns of starch granules. Native starch granules in dry grain from the WT and HP line appear similar in size and shape but differ in surface topography, with the WT showing a smooth granule surface (Fig. 6D) and the HP line showing surface pores (Fig. 6E) also noted in a previous study (Carciofi *et al.*, 2011). The AO line showed very disordered and multilobed morphologies (Fig. 6F), supporting the findings of a previous study (Carciofi *et al.*, 2012) and suggests the presence of abnormal multiple granule initiations typical for high amylose chemotypes, observed in APTS- (8-amino-1,3,6-pyrenetrisulphonic acid, Molecular Probes) stained starch granules [staining performed as in the Supplementary protocol for Fig. S2, available at *JXB* online (Supplementary Fig. S2 available at *JXB* online)] (Wei *et al.*, 2010). On day 2 of germination, WT starch granules were degraded through pits from the inside out, leaving partially hollow granule residues (Fig. 6J) (MacGregor and Ballance, 1980), whereas HP starch granules showed more extensive pits and surface ‘erosion’ possibly from weak spots on the surface (Fig. 6K). The AO starch granules were more resistant to amylolysis (Carciofi *et al.*, 2012) and only a few starch granules showed clear erosion with multiple pits suggesting multiple weak amylolytic attacks (Fig. 6L).

### Histochemistry

**Fig. 7.**
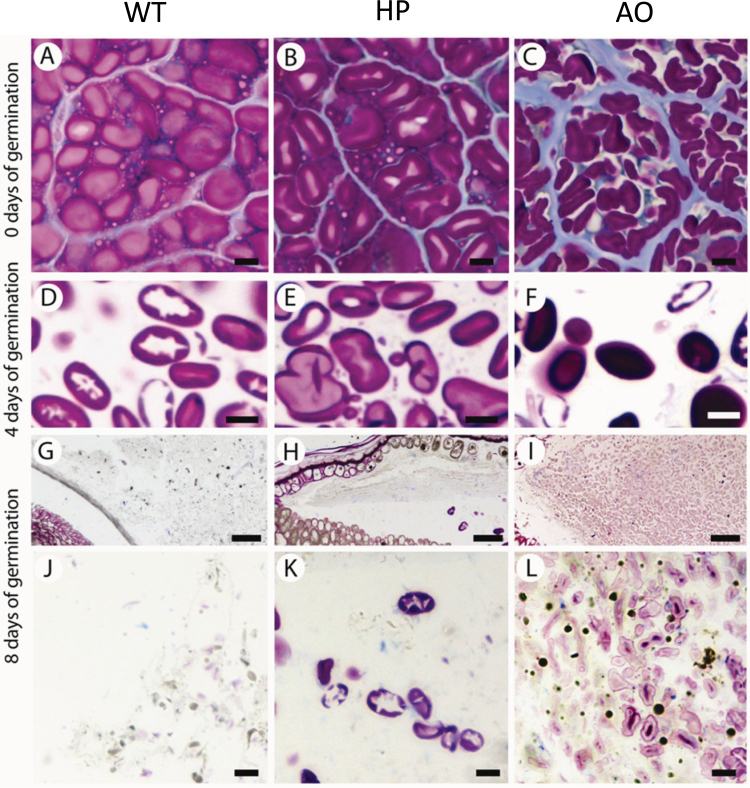
Histochemical analysis of starch degradation by periodic acid–Schiff’s on sectioned endosperm tissue. The sections are counterstained with amido black for proteins. Localization of starch (pink-purple) and protein (blue) near the embryo–endosperm junction at 0, 4, and 8 d germination. Scale bar= 10 μm (A–F, J–L), 50 μm (G–I). A, D, G, and J are from the WT; B, E, H and K are from the HP line; and C, F, I, and L are from the AO line.

The mobilization of starch, proteins, and lipids in the barley endosperm was also investigated by histochemical analyses at different time points (Fig. 7). Besides the red labelling of the starch, PAS also labels other insoluble polysaccharides deposited in the cell walls. Proteins localized in the cytosol and cell wall stain blue with amido black. Post-fixation with heavy metals gives a blackish labelling of the lipophilic substances.

There were remarkable differences during germination among the WT and the two lines with regard to starch granule morphology and rate of degradation, and also differences regarding the appearance of insoluble carbohydrates in general, and the distribution of proteins and lipids. All images are from longitudinal sections through the embryo–endosperm junction of the endosperm.

Before germination, the starch granules from HP endosperm (Fig. 7B) resembled those of the WT (Fig. 7A), both embedded in a densely stained protein matrix, and the endosperm cells are surrounded by thin distinct cell walls. The AO starch granules were sausage-shaped, approximately half the diameter of those of the WT, and proteins are only lightly stained (Fig. 7C).

At 4 d of germination, the starch granules from the WT (Fig. 7D) were highly degraded, with many of the granules with voids in the centre. Proteins were not visible at this stage. Starch granules from the HP line (Fig. 7E) seemed slightly less degraded, whereas in the AO line, grains were densely stained, some without visible signs of degradation (Fig. 7F). This observation is consistent with the starch content data.

More dramatic changes in the PAS reaction were seen within the tissues at 8 d of germination, as starch was almost completely degraded within the entire WT endosperm (Fig. 7G, J). This was also the case for the HP line; however, groups of starch granule remnants can be found in this line (Fig. 7H, K), consistent with the starch content data. In contrast, partially degraded starch granules were present throughout the AO endosperm (Fig. 7I, L). Starch granules were lightly stained with PAS but with a characteristic darker red centre. In the AO line, lipids were also still clearly visible, and are stained black.

## Discussion

Germination is a vulnerable phase of the plant life cycle, in which the small plantlet is particularly sensitive to stress factors. A quick and efficient transfer of energy and biomass from the mother plant is therefore of vital importance for the fitness of a plant. In flowering plants, this transfer is usually mediated via storage compounds in seeds that are optimized for this process. In cereal grains, the dominant storage compound is starch and, in this study, it was demonstrated that bioengineering of the starch structure jeopardizes optimal remobilization of biomass from the grain to the plantlet. Grains were germinated from the barley cultivar Golden promise (WT), which differed in their starch structure with respect to amylose content (Carciofi *et al.*, 2012) and phosphate content (Carciofi *et al.*, 2011). It is important to note that the three types of grains that were compared originate from the same inbred cultivar. There is therefore no difference in genotype among the three grain types, except for the single transgenic gene which causes the altered starch phenotype in the two modified types of grains: AO and HP. Thus, by growing mother plants producing grains under similar conditions, this system allows the study specifically of the effect on germination of the two starch modification types, independent of other variations in genotype or environment. The grains were germinated in the dark in ddH_2_O, and therefore the only opportunity for the grain and germinating plantlet system to generate energy is through respiration of storage compounds. However, a major part of the grain storage compounds are also remobilized to support anabolic pathways of the new plantlet. It was observed that total dry weight biomass was removed less efficiently (Fig. 1A, left) and remobilized less efficiently into young plantlets (Fig. 1B, left) from grains with AO starch than from WT grains. Storage starch is remobilized by a series of hydrolytic enzymes, of which the primary ones are α- and β-amylases. It was previously shown that the AO starch type has a much increased resistance to such hydrolysis, and that a large fraction of residual resistant starch remains after hydrolysis *in vitro* (Carciofi *et al.*, 2012). This suggests that the reduced biomass remobilization in AO grains is due to a higher fraction of undigested starch, which was confirmed by measuring residual starch content in the germinating grains (Fig. 2). Phosphorylation of starch may be a catalytic prerequisite for its hydrolysis (Blennow and Engelsen, 2010), and previous studies indicated that the increased phosphate content of the HP grains made the starch granules more vulnerable to premature degradation (Carciofi *et al.*, 2011). Therefore, HP grains could possibly have a more efficient starch remobilization. However, there was no significant difference in biomass remobilization between WT and HP grains (Fig. 1A, B, right panels), demonstrating that the lower phosphate content of WT grains is already optimized and therefore efficient for optimal biomass remobilization during germination.

### Altered starch molecular structure combined with reduced amylolytic activity limits carbohydrate availability during germination and seedling establishment

Starch provides the major carbon source for generating energy (Hong *et al.*, 2012), precursors, and intermediates required during germination and seedling establishment. The time courses of starch degradation found in the AO, HP, and WT starch types differed from the effects anticipated from current knowledge of starch granule degradability of HP and AO starch types. In the early stage of seedling establishment, the WT and HP grains did not show much reduction in the starch content, but it declined rapidly in the mid and late stages of seedling establishment showing sigmoidal rate dynamics. AO grains, on the other hand, exhibited a slow, linear reduction in starch content through the entire time course.

A slower rate of starch degradation was observed in the HP line as compared with the WT line (Fig 2; Supplementary Table S2 available at *JXB* online). This was unexpected since phosphorylation is known to stimulate starch degradation (Blennow and Engelsen, 2010). However, it was also found that amylase activities were significantly lower in HP and AO grains than in WT grains (Fig. 4), which could explain such a contrary effect. Similarly for the AO line, this finding is in agreement with the present data showing that the reduced amylase activities in this line result in slower degradation of starch. However, such a reduction is also likely to be affected directly by starch structure, as indicated by previous studies, where the direct effects of the altered starch structure in the AO line and the WT were studied by hydrolysis of purified starch at identical amylase activity levels (Carciofi *et al.* 2012).

The initial high rate of degradation in the AO line was not as deduced from its hydrolytic resistance *in vitro* (Carciofi *et al.*, 2012). One possible explanation could be that this initial susceptibility can be attributed to the high effective surface area of these starch granules compared with the WT and HP granules, as evident from SEM (Fig. 6D–F). The surface area is important for the catalytic rate (Yu *et al.*, 1996; Kim *et al.*, 2008; Tahir *et al.*, 2010; Shrestha *et al.*, 2012). Over the entire germination period, the dynamics of starch degradation are lost, showing virtually linear and slow progress of degradation. This effect is expected as a direct effect of the highly compact structure of high amylose starch granules (Shrestha *et al.*, 2012) and, later in the course of germination, by re-organization of the fragmented starch granule residual (Szczodrak and Pomeranz, 1991; Shrestha *et al.*, 2010).

In conclusion, these data suggest that the modified starch structure in the AO and HP lines affects carbohydrate remobilization, not only directly by changes in starch enzyme interactions, but also indirectly through reduced activities of amylases during grain germination and seedling establishment.

### Alternative compensating pathways for carbon utilization in the germinating grain

The demonstrated perturbations in barley seedling starch metabolism are probably compensated by alternative pathways. Hence, utilization of cell wall and protein components was investigated. (1,3;1,4)-β-d-glucan (BG) and, to a lesser extent, arabinoxylan are the main cell wall components in a barley grain (Fincher, 2011). BG declines during germination (Morrall and Briggs, 1978; Etokakpan and Palmer, 1994; Nanamori *et al.*, 2011). Even though BG declined dramatically over the time course, no significant differences in the level of reduction of BG was found among the WT, AO, and HP lines, suggesting that differences in starch utilization are not compensated by BG degradation. The similar levels of arabinose in WT, HP, and AO grains supports that arabinoxylan is also degraded to the same extent. Sugars resulting from cell wall dissolution are indicated to contribute energy for the young seedling (Fincher, 2011) and play a major role as mechanical support during grain filling, and sugar degradation stimulates amylase diffusion in the endosperm. In conditions where carbohydrate is scarce, proteolysis is induced (Brouquisse *et al.*, 1998) and protein can act as an alternative respiratory substrate (Araújo *et al.*, 2011). The present data support this redirection; in the initial stages where the carbon availability from starch degradation is restricted in the HP line, greater protein degradation was observed. In the AO line, the ample availability of carbon from starch degradation in the initial stages is balanced by the low protein degradation observed. Thereafter, a rapid decline in the protein content was observed along with the slow degradation of starch, indicating that protein may act as an alternative respiratory substrate.

In summary, low starch carbon availability is compensated by re-directing the metabolism to protein degradation. These results show that perturbations of starch metabolism in a seedling can affect other metabolic fluxes, in this case protein metabolism, providing essential carbon without severely endangering the seedling’s survival.

### Enzyme and sugar dynamics

Polysaccharide and protein degradation are directly and metabolically linked to sugar metabolism, and the soluble sugar levels increase dramatically upon germination. In the present study, there was no significant difference in total soluble sugar levels in the initial stages of germination between the WT and HP, while AO showed slightly higher levels of sucrose, fructose, and glucose. There have been extensive studies done to establish the mechanism of regulation by sugars and GA in barley (Perata *et al.*, 1997), and recent studies have been performed on the convergence of GA and sugar starvation signals to induce α-amylase expression in barley (Hong *et al.*, 2012). However, no consequent correlation of reduced α-amylase activity with increased sugar levels was seen in the present study. Sugar levels were slightly higher in the AO line than in the WT up to 4 d, but then in the mid and late stages of seedling establishment, sugar levels were much lower in the AO line than in the WT (Fig. 3), whereas α-amylase activity remained much lower than in the WT (Fig. 4). The glucose level in the HP line is higher than in the WT at day 8 (Fig. 3), but α-amylase activity is much lower in the HP line than in the WT already earlier in germination (Fig. 4). Therefore, altered sugar levels do not seem to be the direct determining factor in the effect of altered starch structure on reduced α-amylase activity in the AO and HP grains throughout all levels of germination and seedling establishment.

Endogenous α-amylases are the most abundant hydrolases and they play a central role in the mobilization of starch during germination and seedling establishment (Smith *et al.*, 2005; Hong *et al.*, 2012). The activity of α-amylase was absent in the extracts of pre-germinated dry grains of all three lines, but its activity rapidly appeared and increased as the process of germination occurred. In both the HP and the AO lines, α-amylase activity was distinctly suppressed. Similar results were observed in wheat, where the starch phosphorylator GWD was down-regulated (Ral *et al.*, 2012). An increase in α-amylase activity was observed with the down-regulation of GWD, which complements the observations where overexpression of GWD in the HP lines resulted in reduced α-amylase activity.

β-Amylase, which is deposited in the starchy endosperm during grain development of barley (MacGregor *et al.*, 1971; Hara-Nishimura *et al.*, 1986; Zeeman *et al.*, 2010), has a limited role in native starch degradation (Dunn, 1974). The level of total β-amylase activity at the beginning was very low in the AO line due to low deposition during development of the grain. Total β-amylase activity was generally suppressed in both HP and AO lines throughout germination and seedling establishment. The HP line showed the same level of total β-amylase activity as the WT initially, but remained lower throughout seedling establishment stages as it cannot completely degrade phosphorylated starch. Even though total β-amylase activity was significantly different in the HP line as compared with the WT and AO lines, the maltose levels in the mid and late stages of seedling establishment were similar to that of the WT. This did not affect starch degradation, supporting that the hyperphosphorylated starch in the HP line was easily attacked by amylases, as expected (Blennow and Engelsen, 2010).

Direct dephosphorylation of starch by the two chloroplastic phosphoglucan phosphatases SEX4 and LSF2 is important for degradation of transitory starch in the leaves of *Arabidopsis* (Kotting *et al*., 2009; Santelia *et al.*, 2011) and maize (Weise *et al.*, 2012), and reduced activity of either of the two enzymes leads to accumulation of transitory starch in leaf chloroplasts. To the authors’ knowledge, no studies have identified whether these phosphatases, or any homologues thereof, are involved in the degradation of storage starch in germinating barley grains or any other starchy cereal grain type. Neither has anyone identified or cloned SEX4 or LSF2 in barley. Degradation of cereal grain storage starch during germination and seedling establishment is a very different process from the diurnal accumulation and degradation of starch in leaves. During germination and seedling establishment, most amylolytic enzymes are exported from the scutellum and aleurone cells into the endosperm to degrade starch granules. In *Arabidopsis*, SEX4 and LSF2 are intracellular proteins located in the chloroplasts of leaf cells (Kotting *et al*., 2009; Santelia *et al.*, 2011). However, the possibility that phosphoglucan phosphatases could be accumulated in the endosperm during cereal grain endosperm development to play a role later during germination and seedling establishment cannot be excluded. The barley genome contains two putative homologues of SEX4 and LSF2, which were found to be expressed in barley grains (Supplementary Fig. S4 available at *JXB* online). However, there was no significant difference between expression levels of either of these two genes among HP and WT grains, indicating that the storage starch phosphorylation level does not affect the expression of these putative phosphoglucan phosphatases in grains.

It was hypothesized that the differences in germination metabolism between the three starch types can be attributed solely to the starch structure. However, the data indicate that the pleiotropic metabolic effects in the modified lines, in particularly indirect effects on amylase activity, also result in different sugar levels in the grain and different amylolytic capacities. Thus, the combined effects of starch granule degradability and amylolytic activity levels were translated into vastly different compensatory carbon flows in the different lines. The differential starch granule degradation patterns observed by microscopy were possibly directly attributed to different starch granule susceptibility towards hydrolytic attack as initially directed by granule topography and at later stages by the inner morphology. These interaction effects are complex and depend on various factors such as surface pores on the starch granule (Li *et al.*, 2012), adsorption of amylase by the starch granule (Leloup *et al.*, 1992), the effective surface area of the starch granule available for enzyme attack (Kim *et al.*, 2008; Tahir *et al.*, 2010; Shrestha *et al.*, 2012), long-range structural effects controlling enzyme diffusion to starch granule surfaces (Shrestha *et al.*, 2012), and A-, B-, and Vh-type starch crystalline polymorphs (Jane *et al.*, 2003).

The three isogenic barley models provide very distinctly different starch granule structures with expected different susceptibility towards hydrolysis. SEM data support the differential degradation pattern observed; WT starch granules were degraded through pits from inside out leaving partially hollow granule residues, indicating that the inside of the granule is more susceptible than the flat surface, while HP starch granules with surface pores showed more extensive pits and surface erosion. This can be interpreted as enzymatic attack by surface erosion, indicating that the hyperphosphorylation has disrupted the starch granular surface which was more accessible and eroded. The AO starch granules were more resistant, with only a few starch granules degraded with multiple pits, suggesting multiple weak amylolytic attacks.

For the WT and HP lines, iodine complexation as evaluated from the OD 620/550 were constant, demonstrating that there is no obvious selective degradation of amylose and amylopectin as the granules were hydrolytically eroded. For the AO line, the decrease in OD 620/550 is due to multiple hydrolytic attacks of amylases on the rough granules as supported by TLC and microscopy. Such differences in starch granule degradation inevitably lead to different malto-oligosaccharide degradation products with effects on metabolism, as demonstrated in this work.

The observations by microscopy are consistent with the above investigations and have shown the resistive nature of the AO starch granules to degradation. The disappearance of the starch granules seen at the embryo–endosperm junction in the WT and to a lesser extent in HP grains is indicative of their degradability, while lightly stained starch granules with a characteristic darker red centre still existed in the AO line, indicating the combined effects of reduced amylase activity and increased resistance or susceptibility, respectively, for AO and HP starches, towards hydrolysis.

In summary, the present data show that starch bioengineering affects biomass remobilization during germination and seedling establishment, most probably through the combination of direct effects on the starch granule and molecular structure and the indirect effects on amylase activities. This opens up new perspectives on the importance of fine-tuning starch bioengineering to affect the survival and establishment of seedlings directly or indirectly.

## Supplementary data

Supplementary data are available at *JXB* online,

Figure S1. Different stages of seedling growth in the course of germination used for the study.

Figure S2. Confocal laser scanning microscopy (CLSM) optical section of purified WT and AO starch granules to investigate multiple hila.

Figure S3. Thin-layer chromatography of glucan fragments.

Figure S4. qPCR of putative phosphoglucan phosphatase gene expression in barley grains.

Table S1. Values and statistical significance of grain reserve components and amylolytic enzyme activities are presented as mean±SE of three replicates.

Table S2. Values and statistical significance of soluble sugars are presented as mean±SE of three replicates.

Protocols for Figures S1–S4.

Supplementary Data
