# Supplementary material for: Starch bioengineering affects cereal grain germination and seedling establishment
Source: J Exp Bot. 2014 Mar 18;65(9):2257–70. doi: 10.1093/jxb/eru107 (PMC4036499; doi:10.1093/jxb/eru107)
Supplement: Supplementary Data [file supp_65_9_2257__index.html]

Starch bioengineering affects cereal grain germination and seedling establishment — Starch bioengineering affects cereal grain germination and seedling establishment — Supplementary Data 

# Starch bioengineering affects cereal grain germination and seedling establishment

## Supplementary Data

Data files

**Files in this Data Supplement:**

- Supplementary Data - Supplementary Data
