# Supplementary material for: Starch bioengineering affects cereal grain germination and seedling establishment
Source: J Exp Bot. 2014 Mar 18;65(9):2257–70. doi: 10.1093/jxb/eru107 (PMC4036499; doi:10.1093/jxb/eru107)
Supplement: Supplementary Data [file supp_eru107_jexbot116277_file001.pdf]

Starch bioengineering affects cereal grain germination and seedling establishment

Shahnoor S. Shaik [1], Massimiliano Carciofi [1,2], Helle J. Martens[1], Kim H. Hebelstrup[2†*], Andreas Blennow[1†*]

Supplementary data

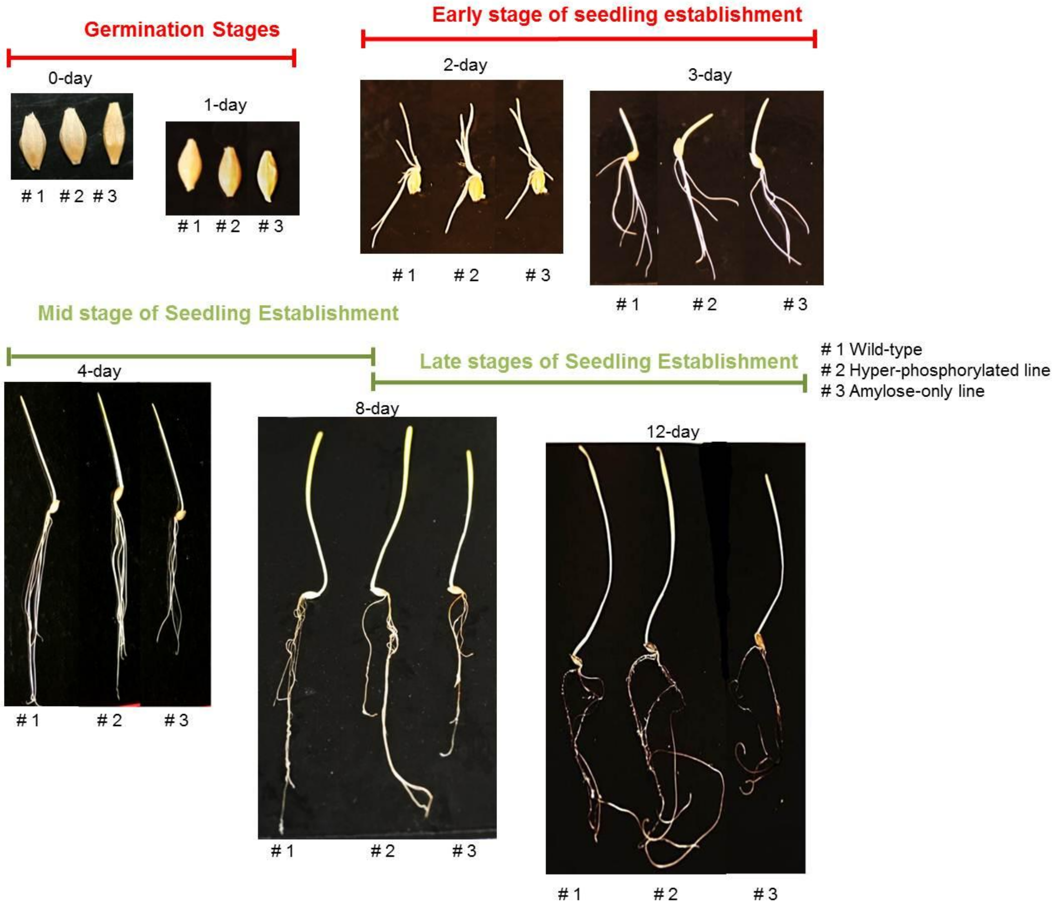

Fig. S1 Different stages of seedling growth in the course of germination used for the study. Grains of the wild type (WT), the hyper-phosphorylated (HP) and the amylose-only (AO) line were germinated in sealed plastic containers on filter paper moistened with autoclaved ddH_2O for one, two, three, four, eight and twelve days. The germination was conducted in the dark in a growth cabinet at a constant temperature of 23°C. There was no difference in the growth of seedlings among WT, HP and AO in the early phase. But the AO seedling growth (#3) has slowed down in the late phase.

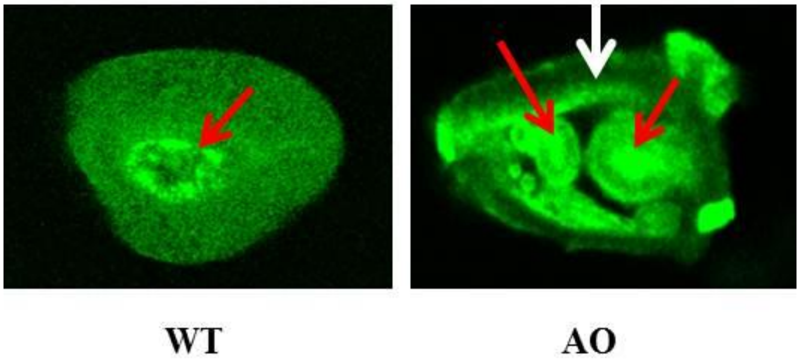

Fig. S2 Confocal Laser Scanning Microscopy (CLSM) optical sections of purified WT and AO starch granules to investigate multiple hila. Starch granules were labeled with the fluorophore APTS. The CLSM instrument (TCS SP2, Leica Microsystems, Wetzlar, Germany) was equipped with an argon laser, the following objective: 40X plan apo/ 1.25–0.75 oil CS, with following filter setting was used: excitation wavelength: 488 nm, beam splitter: TD 488/543/ 633, light was detected at the interval from 500 to 535 nm. AO starch granule showed multiple hila (red arrows) indicating multiple granule initiation and (white arrow) pointing the sac-like structure covering the small granules while WT showed one large starch granule with a single hilum.

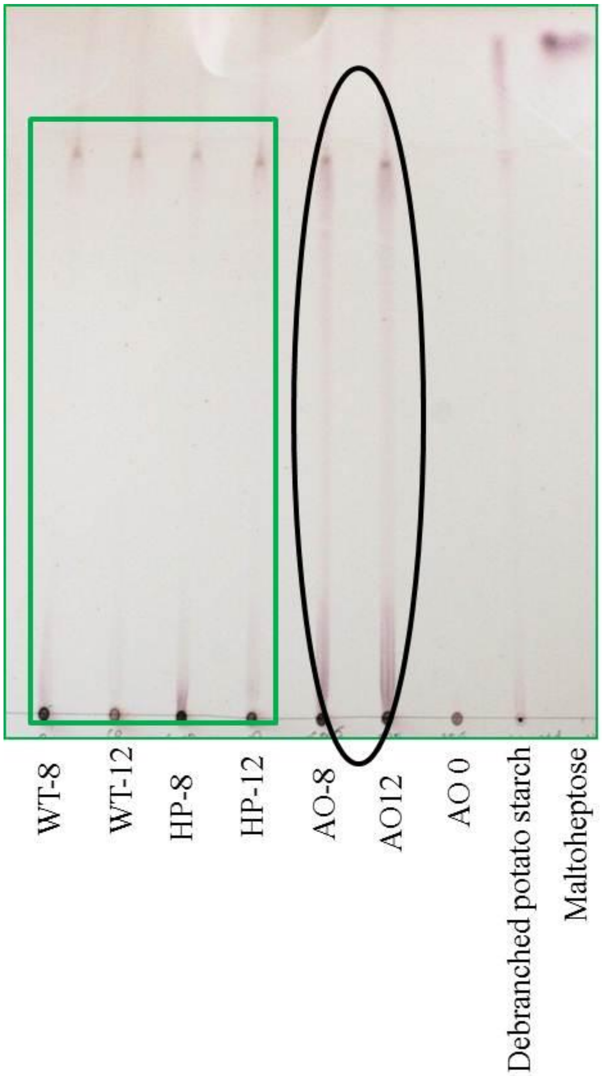

Fig. S3 Thin Layer Chromatography (TLC) of glucan fragments. The grain content was analysed for malto-oligosaccharides. A distinct tailing was observed on the TLC plate in the day eight and twelve samples of AO indicated in “black oval”, which was not observed in the WT and the HP lines indicated by “green rectangle”, supporting the presence of short chain glucans in the residual AO grain generated by multiple attacks on the rough surfaces of the AO starch granules. AO zero day sample, debranched potato starch and maltoheptose were used as controls.

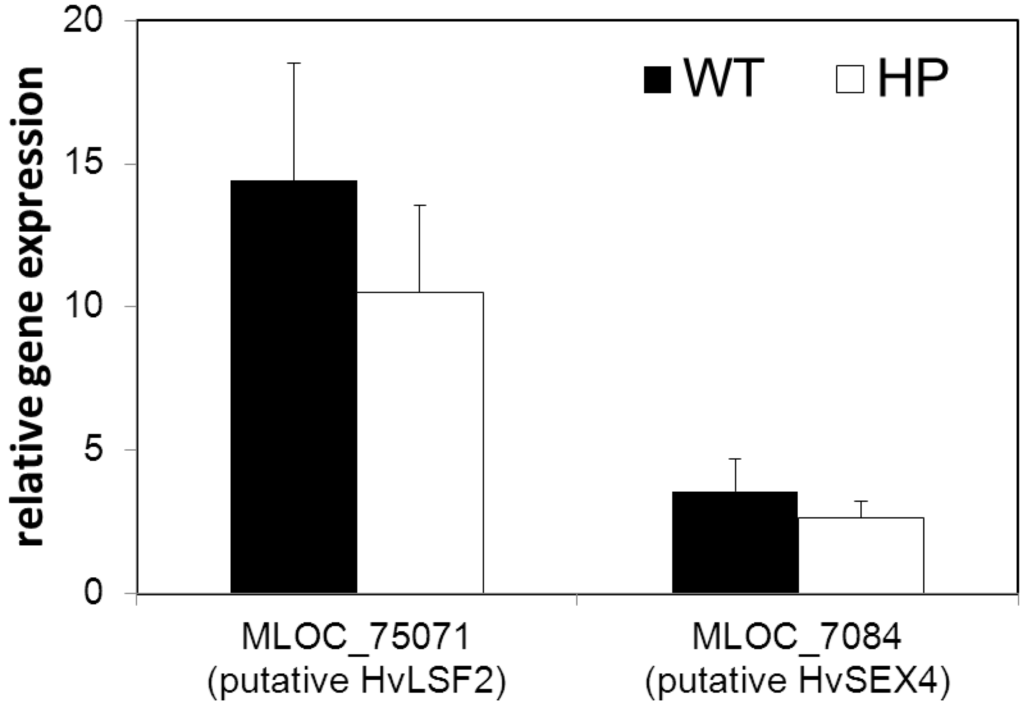

Fig. S4 By blasting the Arabidopsis LSF2 and SEX4 cDNA sequences against the barley genome (http://plants.ensembl.org/Hordeum_vulgare), we identified two (and only two) putative barley phosphoglucan phosphatases: MLOC_75071 with 77% similarity to LSF2 and MLOC_7084 with 61% similarity to SEX4 at the predicted amino acid sequence level. Both of these genes were demonstrated to be expressed in imbibed barley grains by qPCR (see protocol below). However, there was no significant difference in expression levels of either of the two genes between the HP and WT line (Student’s t-test). Gene expression was measured in three biological replicates each with three technical replicates. Bars represent standard errors.

Line	Days after germination	Starch (relative %)	BG (relative %)	Protein (relative %)	α-amylase activity (ceralpha Units/g flour)	β-amylase activity (megazyme Units/g flour)
WT						
	0	100	100	100	1.6±1.6	48.4±3.9
	4	94.0±7.5	44.2±2.0	83.5±1.9	233.4±2.8	65.3±3.1
	8	31.8±1.9	25.3±14.1	76.9±2.0	249.3±1.5	58.2±0.8
	12	28.9±4.3	17.3±10.0	79.3±0.7	67.0±1.9	7.1±0.3
HP						
	0	100	100	100	0.5±0.4	47.1±4.0
	4	98.5±2.1	34.3±5.4	66.3±1.4**	137.0±5.9*	38.1±3.8**
	8	48.7±0.5**	19.1±1.4	61.9±0.4**	134.1±9.5**	32.5±2.0**
	12	30.9±11.9	11.7±1.9	63.9±2.7	27.8±1.4**	7.8±0.9
AO						
	0	100	100	100	0.0±0.3	17.1±0.6**
	4	81.7±0.3	29.9±3.8	88.0±1.5	178.0±3.9	15.6±1.1**
	8	69.1±14.1*	16.6±3.8	67.7±0.3**	104.7±1.1**	5.5±0.3**
	12	56.6±1.2*	23.5±0.1	51.7±1.7**	18.9±1.7**	2.5±0.3**

Supplementary Table S1: Values of grain reserve components and amylolytic enzyme activities are presented as means ± SE of three replicates. Values that are significantly different from WT by one way ANOVA statistical tool embedded into SigmaPlot 12.0 are indicated: * denotes $P < 0.01$ and ** denotes $P < 0.001$.

Line	Days after germination	Total soluble sugars (% DW)	Sucrose (% DW)	Glucose (% DW)	Fructose (% DW)	Trehalose (% DW)	Maltose (% DW)	Arabinose (% DW)	Ribose (% DW)
WT									
	0	4.2±0.3	3.4±0.2	0.1±0.0	0.1±0.01	0.03±0.00	0.2±0.01	0.3±0.01	0.1±0.01
	1	8.3±0.2	6.4±0.1	0.5±0.0	0.2±0.01	0.01±0.00	0.4±0.01	0.3±0.00	0.3±0.01
	2	16.0±0.8	6.7±0.2	2.9±0.1	0.5±0.01	0.01±0.00	4.6±0.30	0.6±0.01	0.7±0.01
	3	36.9±1.0	7.8±0.0	8.3±0.0	0.5±0.00	0.01±0.00	18.1±0.67	1.2±0.01	0.9±0.00
	4	38.6±2.7	5.1±0.2	13.4±0.5	1.2±0.06	0.03±0.00	15.8±0.86	1.5±0.12	1.5±0.03
	8	69.3±4.2	15.0±0.7	23.2±0.6	2.7±0.16	0.22±0.02	21.4±1.20	5.2±1.19	1.5±0.04
	12	42.1±2.2	9.5±0.4	16.4±0.6	2.8±0.19	0.32±0.00	7.7±0.14	3.5±0.13	1.7±0.10
HP									
	0	4.4±0.4	3.5±0.2	0.1±0.0	0.2±0.01	0.03±0.00	0.3±0.06	0.2±0.01	0.1±0.00
	1	7.0±0.5**	5.5±0.3(*)	0.4±0.0**	0.2±0.01	0.01±0.00*	0.3±0.02(*)	0.3±0.01	0.3±0.01
	2	16.9±0.5	6.3±0.0(*)	3.4±0.0**	0.5±0.01	0.01±0.00	5.6±0.24	0.5±0.01(*)	0.6±0.00(*)
	3	36.3±0.8	7.7±0.0	8.6±0.0	0.6±0.06*	0.02±0.00	17.3±0.49	1.1±0.01*	0.9±0.00
	4	43.2±4.5	6.4±0.3(*)	12.6±0.5	1.1±0.06	0.05±0.00	21.0±2.2	1.1±0.05(*)	0.9±0.08*
	8	82.8±6.8	16.2±1.0	35.3±1.1**	2.3±0.06	0.31±0.02(*)	22.1±2.4	5.8±0.15	0.6±0.04*
	12	37.4±3.0	8.1±0.5(*)	15.0±0.5	2.9±0.16	0.37±0.02	7.2±0.74	2.4±0.11	1.3±0.01(*)
AO									
	0	8.5±0.9*	6.2±0.5**	0.2±0.0**	1.3±0.08**	0.08±0.00*	**0.0**(*)	0.3±0.02(*)	0.4±0.03*
	1	9.8±0.1**	9.2±0.2**	0.5±0.0**	0.5±0.02*	0.01±0.00**	0.2±0.00(*)	0.3±0.01	0.3±0.01
	2	20.7±0.5*	10.8±0.1**	4.1±0.0**	1.0±0.01**	0.03±0.00**	4.8±0.13	0.5±0.01(*)	0.9±0.00**
	3	44.2±1.7*	12.3±0.8**	11.6±0.2*	0.8±0.01**	0.04±0.00**	17.8±1.11	1.1±0.01**	1.1±0.02*
	4	49.4±3.7	6.9±0.3(*)	16.5±0.2	2.2±0.15*	0.02±0.00	18.8±1.78	2.0±0.10(*)	2.8±0.09*
	8	28.2±1.4**	7.0±0.3**	8.4±0.2**	0.8±0.02*	0.17±0.00	4.5±0.26**	6.2±0.20(*)	1.2±0.06(*)
	12	15.1±0.9**	4.0±0.1**	3.7±0.1**	0.8±0.07*	0.18±0.00*	1.6±0.20**	3.5±0.03**	1.1±0.05(*)

Supplementary Table S2: Values of soluble sugars are presented as means ± SE of three replicates. Values that are significantly different from WT by one way ANOVA statistical tool embedded into SigmaPlot 12.0 are indicated: (*) denotes $P < 0.05$, * denotes $P < 0.01$ and ** denotes $P < 0.001$.

Supplementary protocols

Protocol: Fig. S1

Grains of the wild type (WT), the hyper-phosphorylated (HP) and the amylose-only (AO) line were germinated in sealed plastic containers on filter paper moistened with autoclaved ddH2O for four, eight and twelve days. The germination was conducted in the dark in a CMP 6010 growth cabinet (Conviron Ltd., Winnipeg, Canada) at a constant temperature of 23°C.

Protocol: Fig. S2

Starch granules were prepared for staining as described previously in (Blennow et al., 2003).The sample was immediately mounted on a glass plate for microscopy. The confocal laser scanning microscope (TCS SP2, Leica Microsystems, Wetzlar, Germany) used was equipped with an argon laser, the following objective: 40X plan apo/ 1.25–0.75 oil CS, and a spectral filtering system allowing free selection of which intervals of wavelengths to be detected. The filter setting was as follows: excitation wavelength: 488 nm, beam splitter: TD 488/543/ 633, light was detected at the interval from 500 to 535 nm.

Protocol: Fig. S3

Thin layer chromatography (TLC) was performed on the WT-8, 12; HP-8, 12 & AO- 0, 8, and 12 samples along with the standard consisting of malto heptose and debranched potato starch. The samples were analyzed by TLC using a silica gel 60 F plate (Merck, Darmstadt, Germany) in a solvent system of acetone: ethanol: water (7:4:8) at RT. The migrated products were visualized by heating the plate at 110°C in an oven, after it was dipped in ethanol-sulfuric acid (9: 1).

Protocol: Fig. S4

Grains of HP and WT were imbibed in the dark for 24 hours. Total mRNA isolation, cDNA synthesis and determination of relative gene expression by qPCR were performed as described in (Carciofi et al. 2012). Based on the genomic and cDNA sequences (http://plants.ensembl.org /Hordeum_vulgare) of the putative barley HvLSF2 (MLOC_75071) and HvSEX4 (MLOC_7084) various combinations of primer pairs were designed and tested for qPCR. All primer pairs were designed to span at least one exon-exon junction to be specific for cDNA and

exclude amplification from genomic DNA, and were found to amplify a DNA fragment with the predicted length. The analysis was based on the primer pairs with the best performance, which was: 5’-TTGGCTCACAACCTCAGAAGCCTG-3’ & 5’-GATCAAAGTCTACTGCTGGTCTTCTCATATG-3’ for the putative HvLSF2 and 5’-CCATGGGAGCAGTGTTGACGTACAG-3’ & 5’-CAAGATCTGGATCTTGCTGCAAGCAG-3’ for the putative HvSEX4. Both of the putative phosphoglucan phosphatases contain a transit peptide indicating targeting to plastids as indicated by (http://www.cbs.dtu.dk/services/ChloroP/).
